# Gut microbiota markers in early childhood are linked to farm living, pets in household and allergy

**DOI:** 10.1371/journal.pone.0313078

**Published:** 2024-11-27

**Authors:** Annika Ljung, Monica Gio-Batta, Bill Hesselmar, Henrik Imberg, Hardis Rabe, Forough L. Nowrouzian, Susanne Johansen, Carl-Johan Törnhage, Gunhild Lindhagen, Margareta Ceder, Anna-Carin Lundell, Anna Rudin, Agnes E. Wold, Ingegerd Adlerberth

**Affiliations:** 1 Department of Infectious Diseases, Institute of Biomedicine, Sahlgrenska Academy, University of Gothenburg, Gothenburg, Sweden; 2 Department of Paediatrics, Institute of Clinical Sciences, Sahlgrenska Academy, University of Gothenburg, Gothenburg, Sweden; 3 Statistiska Konsultgruppen, Gothenburg, Sweden; 4 Department of Molecular and Clinical Medicine, Institute of Medicine, Sahlgrenska Academy, University of Gothenburg, Gothenburg, Sweden; 5 Pediatric Clinic, Skaraborg Hospital, Lidköping, Sweden; 6 Pediatric Clinic, Skaraborg Hospital, Skövde, Sweden; 7 Pediatric Clinic, Skaraborg Hospital, Falköping, Sweden; 8 Pediatric Clinic, Vara Clinical Center, Vara, Sweden; 9 Department of Rheumatology and Inflammation Research, Institute of Medicine, Sahlgrenska Academy, University of Gothenburg, Gothenburg, Sweden; Satyawati College, University of Delhi, INDIA

## Abstract

**Background:**

Children growing up on farms or with pets have a lower risk of developing allergy, which may be linked to their gut microbiota development during infancy.

**Methods:**

Children from the FARMFLORA birth cohort (N = 65), of whom 28 (43%) lived on a dairy farm and 40 (62%) had pets, provided fecal samples at intervals from 3 days to 18 months of age. Gut microbiota composition was characterized using quantitative microbial culture of various typical anaerobic and facultatively anaerobic bacteria, with colonization rate and population counts of bacterial groups determined at the genus or species level. Allergy was diagnosed at three and eight years of age by experienced pediatricians. Generalized estimating equations were used to identify associations between farm residence or pet ownership, gut microbiota development and allergy. Adjustments were made for important potential confounders.

**Results:**

Growing up on a farm was associated with a higher ratio of anaerobic to facultative bacteria in the first week, smaller *Escherichia coli* populations in colonized children in the first months of life and less frequent colonization by *Clostridioides difficile* at 12 months of age. Having pets in the household was associated with more frequent colonization by *Bifidobacterium*, *Lactobacillus* and *Bacteroides* in the first months. A higher ratio of anaerobic to facultative bacteria at one week of age, early colonization by *Bifidobacterium*, *Lactobacillus* and *Bacteroides*, and reduced carriage of *C*. *difficile* at 4–12 months of age all correlated negatively with subsequent allergy diagnosis.

**Conclusions:**

Our findings indicate that lower rates of allergy in children growing up on farms or with pets may be related to early establishment of typical anaerobic commensals in their gut microbiota. However, further studies are needed to validate our observations in this small birth cohort study.

## Introduction

Children growing up on farms have low rates of allergic diseases [1, 2]. One of several possible explanations may relate to early contact with microbes in the farming environment [3], which may become established in the infant’s commensal microbiota and stimulate programming of the immune system towards a tolerogenic phenotype during the crucial early development window [4]. However, detailed studies comparing the gut colonization patterns of farmers’ infants and non-farmers’ infants from the very first weeks of life are lacking. By one year of age, farmers’ infants have a more mature gut microbiota than infants from non-farming families, as assessed using 16S rRNA gene sequencing [5], and the gut microbiota of farm children is enriched in certain putative *Eubacterium* species at 5–13 years of age [6]. Furthermore, gut microbiota maturation by one year of age correlates negatively with the development of asthma or allergy [5, 7–9] and the effect of certain farm exposures on hay fever is mediated in part by microbiota richness at one year of age [10]. Children who are growing up with pets are also less likely than others to develop allergy [11–13], possibly through similar mechanisms.

Gut microbiota acquisition and maturation represent a sequential process. Facultative anaerobic bacteria (“facultatives”), i.e. bacteria that prefer an oxygen-rich milieu but also replicate under anaerobic conditions, are the first to establish in the gut. These include staphylococci, enterococci, *Escherichia coli* and other *Enterobacteriaceae* species. As they thrive and reach high population counts, they gradually consume most of the oxygen in the neonatal gut. Obligate anaerobic bacteria (“anaerobes”) cannot utilize oxygen, and while some anaerobes can tolerate low levels of oxygen, many die upon contact with air. Thus, colonization by anaerobes starts with relatively oxygen-tolerant groups such as bifidobacteria, *Bacteroides* and certain *Clostridium* species. Subsequently, oxygen-sensitive genera are progressively established, forming a complex, highly anaerobic gut microbiota [14]. In this competitive milieu, facultative bacteria thrive less well, and their population levels decline [15–17]. The anaerobe *Clostridioides difficile*, which is a common gut colonizer in infants, is also outcompeted by a complex anaerobic microbiota [18]. Thus, a mature gut microbiota is characterized by a high ratio of anaerobes to facultatives, low population counts of various facultative anaerobes, and a paucity of *C*. *difficile*.

The FARMFLORA birth cohort included children from dairy farms and rural control children in South-West Sweden, many of whom had pets in the household. Consistent with the allergy-protective ‘farm effect’, children from farming families had a lower rate of allergy than the control children at three years of age, although not at eight years [19, 20]. Here, we characterized the gut microbiota composition of FARMFLORA children from birth to 18 months of age using quantitative microbial culture methods [21]. Quantitative microbial culture permitted detection at the genus or species level of many anaerobic and facultative bacterial groups that are characteristic inhabitants of the infant gut, including *Bifidobacterium*, *Bacteroides*, *Lactobacillus*, *Clostridium*, *C*. *difficile* and *E*. *coli* [14]. Importantly, as quantitative information on gut microbiota development in early life is limited [22], it also allowed the absolute abundance (population counts) of bacterial groups to be determined. We then described the patterns of acquisition and development of the gut microbiota in relation to exposure to a farming milieu and living with pets, and with respect to subsequent allergy development.

## Materials and methods

### The FARMFLORA cohort

The FARMFLORA birth cohort included 28 children from dairy farming families and 37 non-farming controls born at term (≥38 gestational weeks) in a rural area of southwest Sweden [19]. Recruitment took place in the period 14^th^ September 2005 - 3^rd^ March 2008. The characteristics of the cohort are shown in Table 1, with further supporting information on antibiotic treatment in S1 Table. The study was approved by the Human Research Ethics Committee, University of Gothenburg, Sweden (Dnr. 363–05, 674–14) and all the parents provided written informed consent.

**Table 1:** Characteristics of the FARMFLORA cohort and comparisons between children exposed, or not exposed, to farm living or pets, and between children with or without allergy at 3 or 8 years of age.

Child characteristics	Total cohort (n = 65)	Raised on a farm			Pets in household^a^			Allergy at 3 years^c,d^			Allergy at 8 years^c,e^		
		Yes (N = 28)	No (N = 37)	p-value	Yes (N = 40)	No (N = 24)	p-value	Yes (N = 11)	No (N = 52)	p-value	Yes (N = 10)	No (N = 38)	p-value
Farming family	28 (43)				21 (52)	7 (29)	0.077	1 (9)	26 (50)	0.02	3 (30)	15 (39)	0.72
Pets in household^a^	40 (62)	21 (75)	19 (53)	0.077				5 (46)	34 (67)	0.30	5 (56)	22 (58)	1.00
Vaginal delivery	55 (85)	25 (89)	31 (84)	0.49	33(82)	21 (88)	0.73	7 (64)	46 (89)	0.06	6 (60)	33 (87)	0.08
Exclusively breastfed at 4 months	34 (52)	17 (61)	17 (46)	0.32	19 (48)	14 (58)	0.45	2 (18)	30 (58)	0.02	3 (30)	23 (61)	0.15
Partially breastfed at 12 months	33 (51)	14 (50)	19 (53)	1.00	17 (44)	15 (62)	0.20	2 (18)	29 (57)	0.04	2 (20)	23 (61)	0.03
Firstborn	29 (45)	10 (35)	19 (51)	0.31	19 (47)	9 (37)	0.60	5 (45)	24 (46)	1.00	7 (70)	17 (45)	0.29
Intrapartum antibiotics^b^	10 (15)	2 (7)	8 (22)	0.17	6 (15)	4 (17)	1.00	4 (36)	6 (12)	0.06	5 (50)	5 (13)	0.02
Antibiotics at 0–6 months^b^	11 (17)	6 (21)	5 (13)	0.50	7 (17)	4 (17)	1.00	1 (10)	10 (20)	0.67	2 (22)	6 (16)	0.65
Antibiotics at 6–12 months^b^	11 (17)	5 (18)	6 (16)	1.00	5 (12)	6 (25)	0.30	3 (27)	7 (14)	0.36	4 (40)	4 (11)	0.047
Antibiotics at 12–18 months^b^	17 (26)	8 (29)	9 (24)	0.77	10 (25)	7 (29)	0.77	5 (46)	12 (24)	0.26	4 (44)	10 (26)	0.42
Girl	32 (48)	18 (64)	14 (38)	0.046	19 (47)	12 (50)	1.00	2 (18)	28 (54)	0.046	4 (40)	21 (55)	0.49
Allergic parent(s)	27 (41)	8 (29)	19 (51)	0.079	12 (30)	14 (58)	0.036	7 (64)	20 (39)	0.18	5 (50)	15 (39)	0.72
Allergy at 3 years^c^^,^^d^	11 (17)	1 (4)	10 (27)	0.017	5 (13)	6 (26)	0.30				5 (50)	4 (11)	0.01
Allergy at 8 years^c^^,^^e^	10 (21)	3 (17)	7 (23)	0.72	5 (19)	4 (20)	1.00	5 (56)	5 (13)	0.01			

Data are presented as numbers (percentages). Comparisons between groups were performed using Fisher’s exact test.

^a^ Information regarding pets (cat and/or dog) in household was missing for one child not raised on a farm.

^b^ See S1 Table for further details on patterns of antibiotic use.

^c^ Data regarding allergy at three and eight years of age in relation to other cohort characteristics have been published previously [19, 20, 23].

^d^ 63 children (97%) were examined for allergy at three years of age.

^e^ 48 children (74%) were examined for allergy at eight years of age.

### Collection of samples and metadata

The gut microbiota of the children was sampled on nine occasions from 3 days to 18 months of age, as previously described [21]. Freshly voided feces were collected by the parents at 1, 2 and 4 weeks and 2, 4, 6, 12 and 18 months, and transported to the laboratory in air-tight bags filled with an anaerobic atmosphere (AnaeroGen Compact, Oxoid Ltd, Basingstoke, UK). In addition, rectal swab samples were collected by staff at the maternity unit at 3 days of age. Both fecal and rectal swab samples were cultured within 24h after collection.

Parental allergy and pets in the household were registered upon inclusion [19]. Feeding pattern was registered by the parents and collected by telephone interviews at infants´ age 6 12 and 18 months [23].

### Gut microbiota composition by quantitative microbial culture

Gut microbiota composition was characterized using quantitative microbial culture, according to a methodology detailed in S1 Appendix, which we have also described previously [21]. In overview, multiple combinations of culture media (9x), growth conditions (2x), and identification methodologies (9x) were used to determine the colonization (presence/absence) and abundance (population counts, CFU/g feces) of various anaerobic or facultative bacterial groups, including many known to dominate in the infant gut microbiota. As expressed at the level of identification (genus/species/other), these comprised: (anaerobes) *Bifidobacterium*, *Bacteroides*, *Lactobacillus*, *Clostridium*, *C*. *difficile*; (facultatives) *Staphylococcus aureus*, coagulase-negative staphylococci (CoNS), *Enterococcus*, *E*. *coli* and other *Enterobacteriaceae* (*Klebsiella*, *Enterobacter*, *Citrobacter*, *Proteus*, *Morganella*, *Raultella*, *Pantoea*, *Hafnia*). Of note, gut colonization by *S*. *aureus* in this cohort has been described in detail elsewhere [24].

In brief, fecal samples were serially diluted in sterile peptone water using a calibrated spoon and each dilution was plated onto a range of non-selective and selective media then incubated under aerobic or anaerobic conditions (S1 Appendix). Bacterial isolates were sub-cultured for purity and identified using biochemical and/or genetic methods (S1 Appendix) [21, 25, 26]. For each sample, the presence/absence of each bacterial group was recorded, and its population count determined from agar plates yielding 10–100 free-lying colonies. The detection limit was 330 (10^2.52^) colony-forming units (CFU)/g feces. Rectal swab samples were cultured on non-selective and selective media as described above although under aerobic conditions only. The presence/absence of facultative bacterial groups was recorded, but due to the non-quantitative nature of swab sampling population counts were not determined.

For each sample, metrics for analysis were the presence/absence of each bacterial group and its population count (CFU/g of feces; measured in colonized children only). In addition, the ratio of population counts of anaerobic to facultative bacteria (anaerobic/facultative ratio) was determined, as a measure of the anaerobic character of the microbiota. The ratio was determined for all samples in which total population counts of both anaerobic and facultative bacteria could be calculated (based on visible growth on the non-selective culture media Brucella blood and Colombia blood agar, respectively).

### Diagnosis of allergy

The children were examined for atopic eczema, asthma, allergic rhinoconjunctivitis, and food allergy by experienced pediatricians at three and eight years of age using set diagnostic criteria (Table 2) [19, 20]. Allergy was defined as having one or more of these diagnoses. As previously reported, 11/63 (17%) children received a doctor’s diagnosis of allergy at three years of age [19]. At eight years of age, 10/48 (21%) children were allergic (Table 2) [20]. Of these, five were also allergic at three years of age and did not grow out of their allergy, and five became allergic after three years of age. Four children who were allergic at age three were no longer allergic at age 8 and two were lost to follow-up.

**Table 2:** Criteria used for the diagnosis of atopic eczema, asthma, allergic rhinoconjunctivitis, and food allergy in the FARMFLORA birth cohort, and numbers of children diagnosed at three and eight years of age.

		Number diagnosed	
Diagnosis	Diagnostic criteria	3 years of age	8 years of age
Atopic eczema	At 3 and 8 years: According to Williams´ criteria [27] or as itching spots on typical locations that had come and gone for at least 6 months, with symptoms present in the last 12 months.	7	5
Asthma	At 3 years: Persistent wheeze for ≥4 weeks or ≥3 episodes of wheezing, with the last episode after 2 years of age, combined with: a) ≥1 minor criterion (symptoms between colds, eczema, allergic rhinoconjunctivitis or food allergy); or b) clinical response to inhaled glucocorticoids or leukotriene antagonists. At 8 years: Wheeze in the last year together with: a) reversal of bronchial obstruction by β_2_ agonist (>12%); b) bronchial hyper-responsiveness upon methacholine challenge (PD 20 <0.6 mg); or c) ongoing asthma treatment with inhaled corticosteroids.	4	4
Allergic rhinoconjunctivitis	At 3 and 8 years: Typical symptoms from eyes and/or nose in the last year when exposed to pollen or animal dander, together with a positive specific IgE-test or skin prick test to the relevant allergen(s).	1	3
Food allergy	At 3 and 8 years: An immediate or late-onset reaction (within the last 12 months) after ingestion of a specific food and improvement upon its elimination from the diet, together with any of the following: a) other signs of allergic disease; b) more than one organ system involved; or c) supported by positive allergy tests, biopsies, or challenge tests.	2	0
Allergy	One or more of the above diagnoses.	11	10

Diagnostic criteria and data regarding allergy at three and eight years of age in the FARMFLORA cohort have previously been published [19, 20].

### Statistical analyses

Comparisons in Table 1 were performed using Fisher’s exact test. Bacterial variables are presented as colonization rates, median population counts in colonized children, and median ratio of anaerobe/facultative population counts. Statistical analyses of gut microbiota composition were performed using generalized estimating equations (GEE) to account for intra-individual correlations in repeated measures data. Continuous variables were analyzed by assuming a log-normal distribution, and binary variables using normal distribution with identity link. A Matern covariance matrix between repeated measures was used. Robust standard errors (HC3 method) were employed to account for violations against distributional assumptions. Time was modelled as a categorical variable, interacting with the group variable of interest to enable a separate evaluation at each specific time point. Results are presented as 1) differences in colonization rates (bacterium present/absent) between groups, and 2) fold changes in bacterial population counts in colonized children and anaerobic/facultative ratios between groups. Analyses were performed both unadjusted and adjusted for the following potential confounders, as applicable: pets in household, growing up on a farm, sex, breastfeeding (proportion of days of any breastfeeding from birth up to sampling), heredity (i.e. allergic parent(s), only considered in analyses regarding allergy). Farm living and pets were mutually adjusted for since farming families tended to keep pets more frequently, and farming [1, 2] and pets [11–13] are both associated with lower risk of allergy. Sex was adjusted for since a high proportion of the farmers´ children were girls (Table 1), and boys are in many studies at higher risk for allergy [28]. Breastfeeding was controlled for since it strongly affects gut microbiota development [29], and is often believed to affect allergy development, although this may be controversial [30]. Heredity was adjusted for in the allergy analyses since it associated negatively with both farm living and pet keeping in the present study (Table 1), and is also a risk factor for allergy [31].

Overall trends in anaerobic/facultative ratios and population counts of different bacterial groups were analyzed as above but including only time (continuous) as explanatory variable.

All statistical tests were performed at the 5% significance level. Statistical analyses were performed by using SAS/STAT^®^ Software, Version 9.4, of the SAS System for Windows (SAS Institute Inc. Cary, NC), and with SPSS Statistics version 29 (IBM Corporation, New York, USA).

The data analyzed in the current study have been published as an independent data publication in a data repository with restricted access [32].

## Results

### Gut colonization pattern

**Fig 1:**
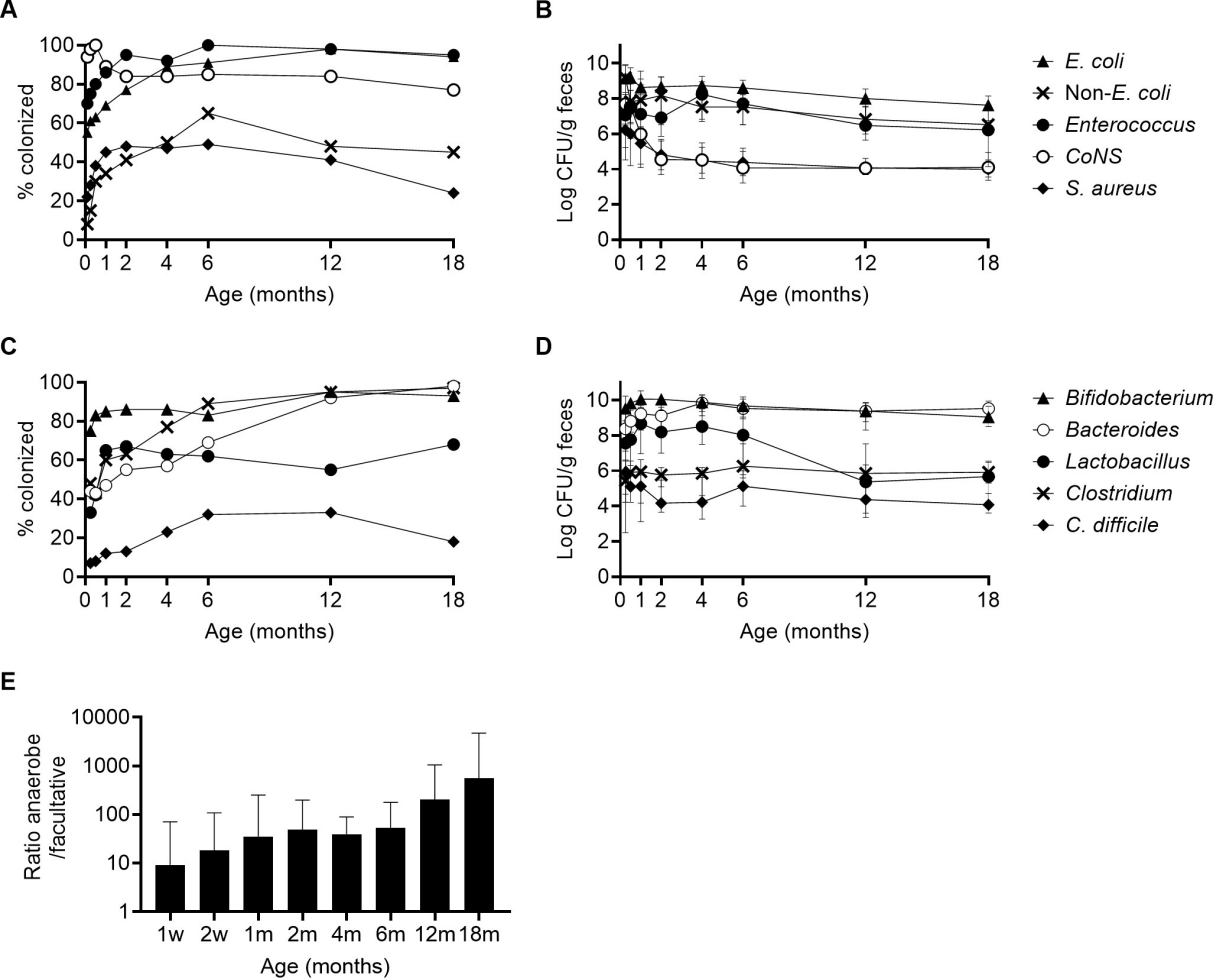
Gut microbiota in the FARMFLORA cohort (N = 65). (**A**) Colonization frequencies (%) of facultative bacteria and (**B**) their median fecal population counts (log CFU/g) in colonized children. (**C**) Colonization frequencies (%) of anaerobic bacteria and (**D**) their median fecal population counts in colonized children. (**E**) Median ratio of anaerobe to facultative fecal bacterial counts (ratio anaerobe/facultative) at different ages. Error bars represent the interquartile range.

Fig 1 shows the pattern of gut colonization by major bacterial groups in the cohort as a whole (N = 65) between 1 week and 18 months of age, and summary statistics for changes over time (bacterial population counts and anaerobic/facultative ratio) are shown in S2 Table. Fig 1A shows colonization by major groups of facultative bacteria. Coagulase-negative staphylococci (CoNS) were the most common initial gut colonizers, being found in >90% of the infants on Day 3, followed by enterococci (70% colonized on Day 3, 95% by 2 months) and *Escherichia coli* (55% on Day 3, 90% by 4 months). Other *Enterobacteriaceae* members (denoted as “non-*E*. *coli*”) appeared later and were less prevalent (Fig 1A, S3 Table), as was *Staphylococcus aureus* (Fig 1A). Colonization by all these groups peaked during the first six months, and either remained constant or declined slightly thereafter.

The fecal population counts of the same groups of facultative bacteria in colonized infants are shown in Fig 1B. Counts were typically higher for Gram-negatives (*E*. *coli* and other *Enterobacteriaceae*, up to ~10^9^ CFU/g) than Gram-positives (*Enterococcus*, CoNS, *S*. *aureus*, *up to* ~10^6^−10^8^ CFU/g). However, for all facultatives, population counts declined significantly between 1 week and 18 months of age. In particular, counts of CoNS and *S*. *aureus* in colonized infants decreased sharply during the first months (Fig 1B, S2 Table).

Fig 1C shows colonization by major culturable anaerobic bacteria. *Bifidobacterium* spp. were detected in the gut of ~80% of infants already in the first week after delivery and colonized most infants up to 18 months of age. *Clostridium* and *Bacteroides* spp. were slower to establish but colonized almost all infants by 6 and 12 months of age respectively (Fig 1C). *Lactobacillus* spp. were also common, colonizing >60% of infants by 1 month of age, although colonization rates did not increase further over time (Fig 1C). *C*. *difficile* was detected in ~30% of infants and colonization rates peaked at 6–12 months of age.

Fig 1D shows the population counts of the same groups of anaerobic bacteria in colonized infants. *Bifidobacterium* spp. were the most abundant group of either anaerobic or facultative bacteria in the first 4 months (~10^10^ CFU/g). However, they decreased slowly in abundance after 4 months of age, while *Bacteroides* counts reached ~10^10^ CFU/g by 4 months and remained high thereafter. *Lactobacillus* counts were lower, peaking at ~10^8.5^ CFU/g at 1–4 months, and declined sharply after 6 months of age (Fig 1D, S2 Table). *C*. *difficile* was present at 10^4−5^ CFU/g feces in colonized infants.

The anaerobic fraction of the gut microbiota increased steadily over the first 18 months, indicated by an increase in the ratio of anaerobe to facultative bacterial counts from ~10 at 1 week of age to ~500 at 18 months of age (Fig 1E, S2 Table).

### Farm living and gut microbiota

**Fig 2:**
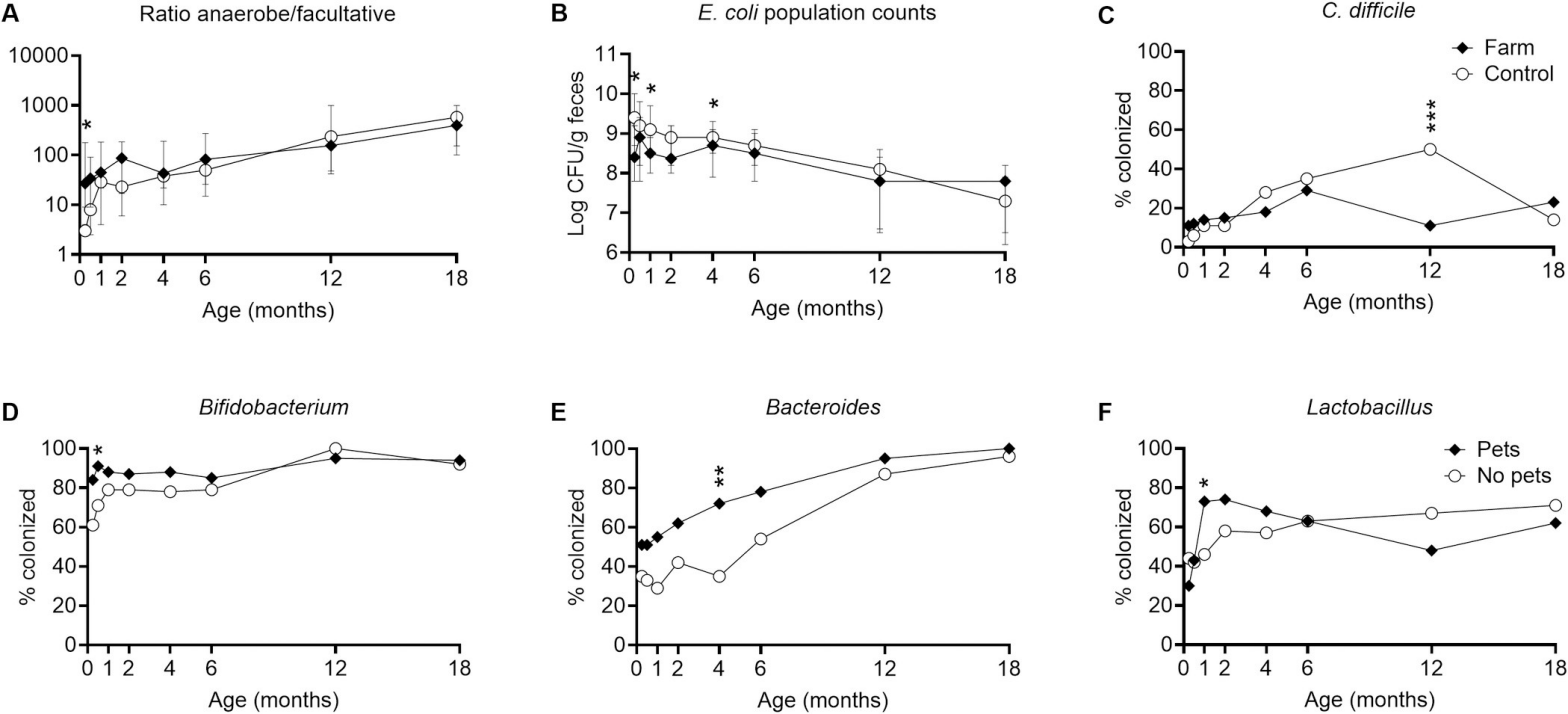
Gut microbiota composition in (A–C) children growing up on a farm vs control children and (D–F) children growing up with pets vs no pets. **A:** Ratio of anaerobe to facultative fecal bacterial counts (ratio anaerobe/facultative) **B:** *E*. *coli* population counts in colonized children. **C–F:** Colonization by *C*. *difficile*, *Bifidobacterium*, *Bacteroides* and *Lactobacillus* spp.. Points are medians (**A–B)** and proportions (**C–F**). Error bars represent the interquartile range. Asterisks show significant differences between groups after adjustment for confounders (farm living adjusted for pets in household, sex, breastfeeding; pets in household adjusted for farm living, sex, breastfeeding). *p<0.05, **p<0.01, ***p<0.001.

Fig 2A–2C show comparisons between farm and control infants regarding gut microbiota characteristics. Significant differences between farming and non-farming infants after adjustment for pet ownership, which was more common on farms, and also for sex and breastfeeding (proportion of days of any breastfeeding up to sampling) are indicated by asterisks. In addition, details of both crude and adjusted associations are shown in S4 Table.

After adjustment for covariates, farm infants had a seven-fold higher ratio of anaerobe to facultative bacterial counts than controls at one week of age (p = 0.020) (Fig 2A), although not at later time points. Hence, when colonized by *E*. *coli*, farmers’ infants had lower counts of this facultative bacterium than controls up to 4 months of age (p = 0.017 at one week; p = 0.030 at 1 month; p = 0.021 at four months) (Fig 2B). Infants growing up on a farm were much less frequently colonized by *C*. *difficile* compared to controls at 12 months of age (p<0.001), although not at other timepoints (Fig 2C). Regarding CoNS colonization rate, farmers’ infants were colonized more frequently at 4 months, but less frequently at 12 months of age (p = 0.029 and p = 0.014 respectively) (S4 Table). Further, when colonized by *Bifidobacterium* and *Lactobacillus*, population counts of these anaerobes were higher at 6 months of age in farmers´ infants than in controls (p = 0.032 and p = 0.027 respectively) (S4 Table).

### Pet exposure and gut microbiota

Fig 2D–2F show colonization by different bacterial groups in infants with or without pets, with significant differences after adjustment for farm living, sex and breastfeeding indicated by asterisks.

After adjustment for covariates, infants with pets acquired *Bifidobacterium* and *Bacteroides* earlier than infants without pets (p = 0.022 at 2 weeks and p = 0.002 at 4 months respectively) (Fig 2D and 2E) and were also more frequently colonized by *Lactobacillus* in the first months (p = 0.045 at 1 month) (Fig 2F). Details of these associations, and also data for other bacterial groups that differed significantly at any time point in either crude or adjusted analysis, are shown in S5 Table. The anaerobe/facultative ratio was non-significantly increased in infants from pet-keeping families at 1 week of age after adjustment (p = 0.088) (S5 Table).

### Gut microbiota and allergy at three years of age

**Fig 3:**
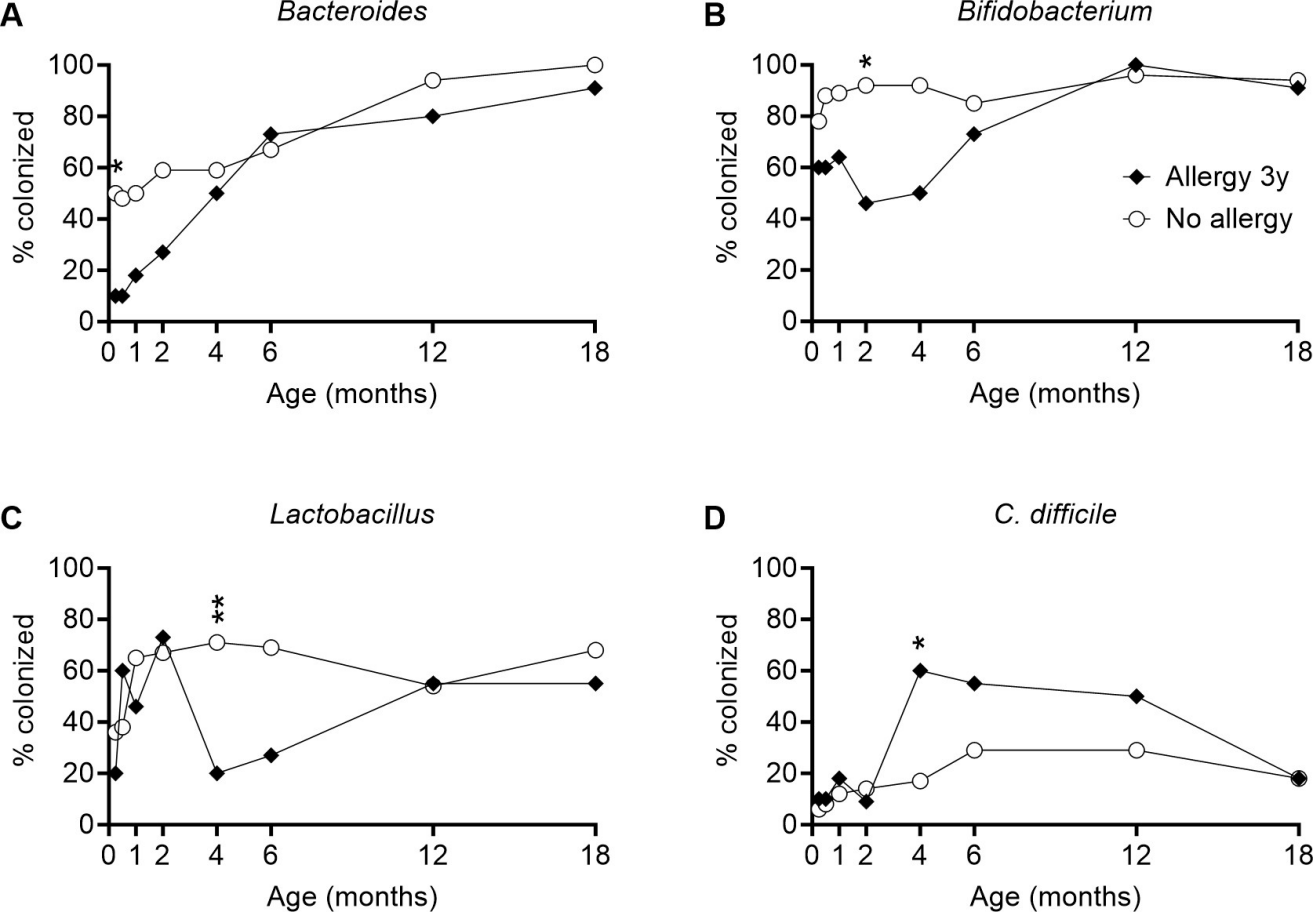
Gut microbiota composition in relation to allergy at three years of age. **A–D**: Colonization by *Bacteroides*, *Bifidobacterium*, *Lactobacillus* and *C*. *difficile*. Points are proportions of infants colonized (**A**–**D**). Asterisks show significant differences between groups after adjustment for confounders (pets in household, growing up on a farm, sex, breastfeeding, allergic parent(s)) *p<0.05, **p<0.01, ***p<0.001.

Next, we investigated the relationship between gut colonization in infancy and later allergy development. Allergy was evaluated at 3 and 8 years of age by clinical assessment (Table 2). Eleven children (17%) were allergic at 3 years of age. Fig 3 shows colonization by certain bacterial groups in infants who were allergic or non-allergic at this age. Significant differences between the groups after adjustment for growing up on a farm, pet exposure, sex, breastfeeding and heredity (allergic parent(s)) are indicated by asterisks.

After adjustment for covariates, allergy at three years of age was associated with delayed colonization by *Bacteroides* (p = 0.031 at 1 week) and *Bifidobacterium* (p = 0.032 at 2 months), and also with less frequent colonization by *Lactobacillus* (p = 0.002 at 4 months) (Fig 3A–3C). Conversely, allergy at three years of age was associated with more frequent colonization by *C*. *difficile* (p = 0.043 at 4 months) (Fig 3D). Allergy at three years of age was also associated with a lower anaerobe/facultative ratio in the first week samples, although not reaching statistical significance after adjustment (p = 0.094), and with more frequent colonization by *Clostridium* spp. (p = 0.033 at 4 months) and CoNS (p = 0.017 and p = 0.036 at 12 and 18 months of age respectively), and higher counts of *S*. *aureus* at 6 months (p = 0.039). Detailed results for bacterial groups associated with allergy at three years of age, in both crude and adjusted analysis, are shown in S6 Table.

### Gut microbiota and allergy at eight years of age

**Fig 4:**
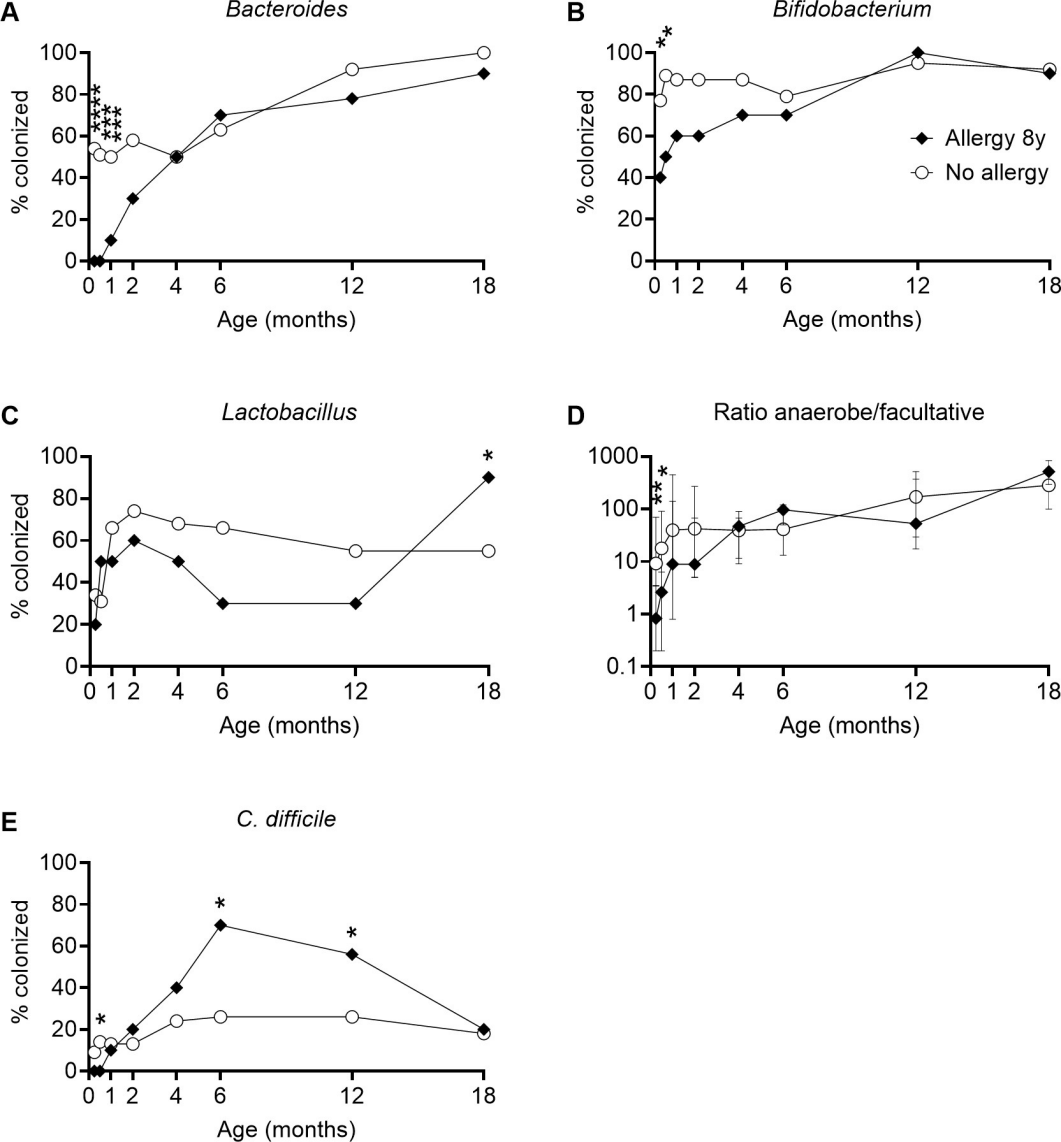
Gut microbiota composition in relation to allergy at eight years of age. **A–C:** Colonization by *Bacteroides*, *Bifidobacterium* and *Lactobacillus* spp. **D:** Ratio of anaerobe to facultative fecal bacterial counts (ratio anaerobe/facultative). **E:** Colonization by *C*. *difficile*. Points are proportions (**A**–**C, E**) and medians (**D**). Error bars represent the interquartile range. Asterisks show significant differences between groups after adjustment for confounders (pets in household, growing up on a farm, sex, breastfeeding, allergic parent(s)). *p<0.05, **p<0.01, ***p<0.001, ****p<0.0001.

Infant gut microbiota characteristics were even more clearly associated with allergy at eight years of age (Fig 4). Ten children (21%) were allergic at this age, of whom 5 were also allergic at 3 years, while 4 children who were allergic at 3 years no longer had an allergy.

After adjustment for the same factors as above, allergy at eight years of age was strongly associated with delayed colonization by *Bacteroides* (p<0.0001 at 1 week, p<0.001 at 2 weeks and p<0.001 at 1 month) and also with delayed acquisition of *Bifidobacterium* (p = 0.046 at 1 week and p = 0.026 at 2 weeks), as well as *Lactobacillus* through most of the first year (although not reaching statistical significance) (Fig 4A–4C). However, *Lactobacillus* colonization was increased in subsequently allergic children by 18 months of age (p = 0.017) (Fig 4C). High anaerobe/facultative ratios at 1 and 2 weeks of age correlated negatively with allergy at 8 years of age (p = 0.007 and 0.047 respectively) (Fig 4D). Allergy at eight years of age was also associated with less frequent initial colonization by *C*. *difficile* (p = 0.049 at 2 weeks), but more frequent colonization by *C*. *difficile* at 6 and 12 months (p = 0.019 and p = 0.049 respectively) (Fig 4E). Detailed results for bacterial groups associated with allergy at eight years of age, in both crude and adjusted analysis, are shown in S7 Table.

## Discussion

In this prospective birth cohort study, we examined the effects of growing up on farms or with pets on the acquisition and development of the gut microbiota during early childhood, as well as associations between the gut microbiota and allergy at three and eight years of age. We observed specific differences in colonization patterns in infants growing up on farms, compared to control infants from the same rural area, as well as specific gut microbiota markers of growing up with pets in the household. Finally, we found quite strong associations between the early colonization pattern and allergy, diagnosed at either 3 or 8 years of age.

While acquisition of the microbiota commences at birth, new species are successively established and the gut microbiota continues to develop and mature, i.e., becomes more diverse and increasingly dominated by obligate anaerobes, during infancy and early childhood [14]. In the present study, we used quantitative culturing of feces to map this process. We did not attempt to cover fully the anaerobic colonization pattern, instead focusing on the major early colonizers of infants [14], which are readily identified by culturing [33, 34]. The development of a complex and more mature gut microbiota was firstly evidenced by a gradual increase in the ratio of anaerobe to facultative bacterial counts in feces, from an average of 10 at 1 week of age to 500 at 18 months of age, which is in line with the results of previous studies [15, 33, 35]. Secondly, a mature anaerobic microbiota can suppress facultative bacteria and reduce their population counts [14, 17, 33] and we accordingly observed that fecal population counts of different facultative bacteria declined significantly between 1 week and 18 months of age in infants colonized by these bacteria. Thirdly, a more mature microbiota counteracts colonization by *C*. *difficile* [18], which is common in young infants. Hence, we observed a decreased carriage rate of *C*. *difficile* after 6–12 months of age.

Among the targeted anaerobes, *Bifidobacterium* spp. were clearly dominant first colonizers, followed by *Clostridium* spp., while *Bacteroides* spp. increased somewhat later and colonized all infants by the end of the first year, at which time their population counts equaled those of bifidobacteria. In addition, lactobacilli appeared early, but colonized only around 60% of the infants during the first year, and their population counts were also lower than for either *Bifidobacterium* or *Bacteroides*, as noted previously [14, 25]. We observed a steep drop in fecal *Lactobacillus* counts between 6 and 12 months of age in colonized infants, which occurred concurrently with the introduction of solid foods and cessation of breastfeeding [23]. This finding has been reported previously by us for other birth cohorts [25, 33], and may relate to major changes in the gut microbiota occurring during weaning.

We observed that certain gut microbiota markers were associated with growing up on a farm. These included a higher anaerobe/facultative ratio in the first week after delivery, lower population counts of *E*. *coli* in colonized infants during the first 4 months, and a markedly reduced carriage of *C*. *difficile* at 1 year of age. All these findings point to a more developed anaerobic gut microbiota in farmers’ infants compared to controls, and were independent of sex, degree of breastfeeding and pet ownership. Other factors known to influence gut microbiota development were similar in children growing up on a farm and control children and could not explain the differences observed, including rates of caesarian delivery [33, 36], exposure to antibiotics [37] and birth order [33, 38]. Notably, two of the microbial markers above differed between farmers´ and non-farmers´ infants already in their first week, which suggests that growing up on a farm affects not only microbiota development towards the end of the first year, as previously shown [5, 10], but also much earlier. This is of interest given that priming of the immune system for tolerance likely starts very early in life, before the atopic march has begun [39]. To the best of our knowledge, this is the first study to demonstrate an association between growing up on a farm and gut microbiota development in the very first weeks of life.

We also observed associations between living with pets and the gut microbiota development of infants, independent of sex, degree of breastfeeding and growing up on a farm. Infants growing up in a home with pets acquired anaerobic bacteria such as bifidobacteria and *Bacteroides* earlier than other infants and were also more frequently colonized by lactobacilli in the first months. A previous study observed that a larger proportion of pet-exposed infants harbored animal-specific *Bifidobacterium pseudolongum* in their gut at 1 month of age than unexposed controls [40]. However, other studies of the gut microbiota in infants in relation to pet exposure have shown inconsistent results [41, 42], and additional studies are needed to confirm our findings.

The most striking observations in the present study were the strong negative associations between being allergic at eight years of age and a high anaerobe/facultative ratio in feces during the first 1–2 weeks after delivery, early colonization with *Bacteroides* and *Bifidobacterium*, and decreased carriage of *C*. *difficile* at 6 and 12 months of age. Similar, but not equally strong associations were observed for allergy at 3 years of age. All these findings suggest that early colonization by obligate anaerobes is linked to protection from allergy development. The link between reduced *C*. *difficile* colonization and protection from subsequent allergy is in line with previous studies [43–45], while the apparent protective effect of early *Bacteroides* colonization is in line with some [5, 46, 47] but not all [48, 49] studies. Also, bifidobacterial colonization has been associated with protection in some studies [46, 50]. We have previously noted that early bifidobacterial colonization in the present cohort was associated with signs of both B- and T-cell maturation [51, 52].

Colonization by *Lactobacillus* at 4 months correlated negatively with allergy at three years of age and was also non-significantly lower during the first year in children who were allergic at eight years of age. Some other studies have also noted negative associations between *Lactobacillu*s colonization and later allergy [53, 54].

As microbiota patterns that were correlated with reduced allergy development were evident already at 1 week of age, very early signals derived from the gut microbiota may be crucial for appropriate immune maturation and protection from subsequent allergy. In accordance, we and others have shown an association between high diversity of the very early gut microbiota and protection against eczema in early childhood [47, 55]. However, since the immune system is transiently activated every time a new bacterium settles in the gut [56], the continuous acquisition of new bacterial species and strains during infancy may provide the repetitive immune stimuli needed to fully promote the development of tolerance against harmless environmental antigens (“allergens”) [39]. This is supported by our findings of positive associations between *C*. *difficile* carriage at 4–12 months of age and subsequent allergy, since *C*. *difficile* carriage indicates gut microbiota immaturity, and also by the results of others linking an immature gut microbiota at one year of age to later allergy [5, 7–9]. A mature microbiota might also produce metabolites that favor tolerance development, e.g., short chain fatty acids. Accordingly, the farm children in FARMFLORA had higher fecal levels of iso-butyric, iso-valeric and valeric acid at three years of age than the rural controls [57]. Higher fecal levels of valeric acid at age 3 were associated with a low rate of eczema at age 8 in this cohort [57], and were a negative predictor of eczema and food allergy in another Swedish cohort [58], while fecal butyrate and butyrate-producing bacteria in the microbiota at 1 year of age were negatively associated with asthma development in the PASTURE study [5].

A limitation of the present study was the relatively small sample size which reduced the statistical power and precision of the analyses and may limit the generalizability of the findings. Also, due to the exploratory nature of this analysis we did not correct for multiple testing. Therefore, further studies are needed to validate our results. The strengths of the study were the thorough and structured clinical examinations for allergy at three and eight years of age, and the immediate culturing of fresh fecal samples at numerous time points.

Bacterial culturing has certain inherent advantages and drawbacks. Culturing has excellent sensitivity for early colonizing anaerobes such as bifidobacteria, lactobacilli, *Bacteroides*, members of *Clostridium sensu stricto* and *C*. *difficile*, as well as facultatives such as *E*. *coli*, *S*. *aureus* and enterococci. It also allows quantification of the population counts of these bacterial groups. In contrast, traditional 16S rRNA gene sequencing only provides information on their relative abundance, although more recent studies may combine 16S rRNA gene sequencing with enumeration of bacterial cells or quantitative PCR to allow absolute quantification [59, 60]. Furthermore, culturing allows us to identify some key bacterial groups at the sub-genus or species level, while 16S rRNA gene sequencing only enables differentiation at the genus or even higher taxonomic levels [34, 61]. The major drawback of culturing is that the fastidious, highly oxygen-sensitive anaerobes which establish in the gut microbiota from weaning and onwards are missed, unless multiple and advanced culture systems are applied [61]. Therefore, we concentrated on the major early anaerobic colonizers that are readily detected by culture in this study.

It is well-established that growing up on a farm strongly protects against allergy development [1, 2], and accelerated gut microbiota maturation may be a contributing factor to this effect [5, 10]. Growing up with pets is also associated with protection from allergy [11–13], but the mechanism for this effect is unclear. In the present study, some gut microbiota markers that were linked to growing up on a farm and/or living with pets were also associated with reduced risk of subsequent allergy, including a higher anaerobic/facultative ratio, earlier colonization by *Bifidobacterium* and *Bacteroides* spp., and reduced colonization by *C*. *difficile*. However, the associations between early gut microbiota patterns and subsequent allergy were clearly stronger than those between farm living or pet exposure and composition of the gut microbiota. Thus, whereas the protective effect against allergy of farm living or contact with pets may, in part, be explained by effects on gut microbial colonization pattern, other factors are also likely to contribute.

## Supporting information

S1 Table Antibiotic treatment of the mothers during delivery and of the children during infancy. (DOCX)

S2 Table Linear trends in population counts of different bacterial groups in colonized children and ratio of anaerobe/facultative bacterial population counts from 1 week to 18 months of age. (DOCX)

S3 Table Colonization frequency and fecal population counts of *Enterobacteriaceae* species in colonized infants at different time-points. (DOCX)

S4 Table Bacterial variables associated with farm living, unadjusted and adjusted for pets in household, sex, and breastfeeding (proportion of days of any breastfeeding up to sampling). (DOCX)

S5 Table Bacterial variables associated with pets in household, unadjusted and adjusted for farm living, sex, and breastfeeding (proportion of days of any breastfeeding up to sampling). (DOCX)

S6 Table Bacterial variables associated with allergy at 3 years of age, unadjusted and adjusted for farm living, pet exposure, sex, breastfeeding (proportion of days of any breastfeeding up to sampling) and heredity (allergic parent(s)). (DOCX)

S7 Table Bacterial variables associated with allergy at eight years of age, unadjusted and adjusted for farm living, pet exposure, sex, breastfeeding (proportion of days of any breastfeeding up to sampling) and heredity (allergic parent(s)). (DOCX)

S1 Appendix Culture media, growth conditions and identification methods used for the isolation and identification of bacterial groups that are commonly detected in the early gut microbiota. (DOCX)
