# Supplementary material for: Gut microbiota markers in early childhood are linked to farm living, pets in household and allergy
Source: PLoS One. 2024 Nov 27;19(11):e0313078. doi: 10.1371/journal.pone.0313078 (PMC11602077; doi:10.1371/journal.pone.0313078)
Supplement: S1 Table — (DOCX) [file pone.0313078.s001.docx]

**S1 Table.** Antibiotic treatment of the mothers during partus and of the children during infancy.

| **Intrapartum antibiotics** | **Numbers treated** | | |
| --- | --- | --- | --- |
| Cefuroxime | 2 | | |
| Cefuroxime plus metronidazole | 2 | | |
| Benzylpenicillin | 1 | | |
| Benzylpenicillin plus cefuroxime | 1 | | |
| Erythromycin | 2 | | |
| Missing data on antibiotic type | 2 | | |
| **Antibiotic treatment of the child during infancy** | **0–6 months** | **6–12 months** | **12–18 months** |
| Ampicillin | 1 | 3 | 1 |
| Amoxicillin | 0 | 0 | 2 |
| Amoxicillin/clavulanic acid | 0 | 0 | 2 |
| Cephalosporin | 1 | 1 | 0 |
| Isoxazolylpenicillin | 0 | 1 | 2 |
| Phenoxymethylpenicillin | 4 | 10 | 13 |
| Trimethoprim | 0 | 0 | 1 |
| Trimethoprim/sulfamethoxazole | 3 | 0 | 4 |
| Missing data on antibiotic type | 2 | 0 | 0 |
