# Supplementary material for: Gut microbiota markers in early childhood are linked to farm living, pets in household and allergy
Source: PLoS One. 2024 Nov 27;19(11):e0313078. doi: 10.1371/journal.pone.0313078 (PMC11602077; doi:10.1371/journal.pone.0313078)
Supplement: S2 Table — (DOCX) [file pone.0313078.s002.docx]

**S2 Table.** Linear trends in population counts of different bacterial groups in colonized children and ratio of anaerobe/facultative bacterial population counts from 1 week to 18 months of age.

|  | 1 week – 6 months | | 1 week – 12 months | | 1 week – 18 months | |
| --- | --- | --- | --- | --- | --- | --- |
| Variable | Change in bacterial population counts per month (95% CI) | p-value | Change in bacterial population counts per month (95% CI) | p-value | Change in bacterial population counts per month (95% CI) | p-value |
| **Facultative bacteria ^a^** |  |  |  |  |  |  |
| *E. coli* | -0.06 (-0.12 to 0.01) | 0.09 | -0.09 (-0.12 to -0.06) | <0.001 | -0.10 (-0.12 to -0.07) | <0.001 |
| *non-E. coli Enterobacteriaceae* | -0.08 (-0.21 to 0.05) | 0.20 | -0.10 (-0.16 to -0.04) | 0.001 | -0.08 (-0.12 to -0.04) | <0.001 |
| *Enterococcus* | 0.09 (-0.02 to 0.20) | 0.10 | -0.08 (-0.12 to -0.03) | 0.002 | -0.08 (-0.12 to -0.05) | <0.001 |
| *S. aureus* | -0.25 (-0.35 to -0.15) | <0.001 | -0.13 (-0.19 to -0.07) | <0.001 | -0.10 (-0.13 to -0.06) | <0.001 |
| *CoNS* | -0.52 (-0.59 to -0.45) | <0.001 | -0.22 (-0.25 to -0.18) | <0.001 | -0.13 (-0.15 to -0.11) | <0.001 |
| **Anaerobic bacteria ^a^** |  |  |  |  |  |  |
| *Bacteroides* | 0.19 (0.10 to 0.28) | <0.001 | 0.06 (0.03 to 0.09) | <0.001 | 0.04 (0.02 to 0.06) | <0.001 |
| *Bifidobacterium* | -0.00 (-0.07 to 0.07) | 0.97 | -0.07 (-0.11 to -0.03) | 0.002 | -0.05 (-0.07 to -0.03) | <0.001 |
| *Lactobacillus* | 0.02 (-0.14 to 0.18) | 0.80 | -0.18 (-0.27 to -0.10) | <0.001 | -0.14 (-0.18 to -0.10) | <0.001 |
| *Clostridium* | 0.15 (0.06 to 0.25) | 0.002 | 0.03 (-0.00 to 0.07) | 0.08 | 0.02 (-0.02 to 0.04) | 0.08 |
| *C. difficile* | 0.12 (-0.05 to 0.30) | 0.16 | -0.01 (-0.08 to 0.07) | 0.89 | -0.02 (-0.06 to 0.02) | 0.33 |
| **Ratio anaerobes/facultatives** (log) | 0.10 (0.04 to 0.15) | 0.001 | 0.09 (0.07 to 0.12) | <0.001 | 0.09 (0.07 to 0.11) | <0.001 |

Statistical analyses were performed using generalized estimating equations (GEE) accounting for intra-individual correlation in repeated measures data. ^a^ Units are log CFU/g feces.
