# Supplementary material for: Gut microbiota markers in early childhood are linked to farm living, pets in household and allergy
Source: PLoS One. 2024 Nov 27;19(11):e0313078. doi: 10.1371/journal.pone.0313078 (PMC11602077; doi:10.1371/journal.pone.0313078)
Supplement: S3 Table — (DOCX) [file pone.0313078.s003.docx]

**S3 Table.** Colonization frequency and fecal population counts of *Enterobacteriaceae* species in colonized infants at different time-points.

|  | **% of infants colonized**  **(median log population counts in colonized infants)** | | | | | | | | |
| --- | --- | --- | --- | --- | --- | --- | --- | --- | --- |
| **Species** | **3 d** | **1 w** | **2 w** | **1 m** | **2 m** | **4 m** | **6 m** | **12 m** | **18 m** |
| *E. coli* | 55.6  ND | 60.7  (9.1) | 63.3  (9.2) | 69.2  (8.6) | 76.6  (8.7) | 89.1  (8.7) | 90.8  (8.6) | 98.4  (8.0) | 93.5  (7.6) |
| *Klebsiella spp.* | 4.8  ND | 11.5  (9.3) | 20.0  (8.2) | 27.7  (7.9) | 28.6  (8.3) | 39.1  (7.3) | 46.2  (7.6) | 32.8  (6.5) | 19.4  (6.4) |
| *Enterobacter spp.* | 1.6  ND | 1.6  (9.5) | 6.7  (7.8) | 7.7  (7.2) | 6.3  (7.8) | 1.6  (7.5) | 9.2  (6.7) | 10.9  (7.0) | 11.3  (6.5) |
| *Citrobacter spp.* | 0 | 0 | 3.3  (7.0) | 3.1  (8.7) | 7.9  (7.5) | 9.4  (7.9) | 23.1  (7.7) | 17.2  (6.5) | 16.1  (6.9) |
| *Proteus spp.* | 0 | 4.9  (5.8) | 3.3  (6.5) | 1.5  (7.5) | 4.8  (8.0) | 7.8  (7.3) | 4.6  (8.4) | 1.6  (7.1) | 0 |
| *Morganella spp.* | 0 | 0 | 0 | 0 | 1.6  (8.9) | 3.1  (8.2) | 1.5  (8.6) | 0 | 0 |
| *Raultella spp.* | 0 | 0 | 0 | 0 | 3.2  (9.0) | 1.6  (7.8) | 3.1  (7.2) | 0 | 0 |
| *Pantoea spp.* | 0 | 0 | 0 | 0 | 0 | 0 | 4.6  (6.5) | 0 | 0 |
| *Hafnia spp.* | 0 | 0 | 0 | 0 | 0 | 0 | 0 | 1.6  (8.2) | 1.6  (6.5) |

Only bacterial genera isolated from more than one sample are included in the table. Abbreviations: ND, Not determined. Quantitative cultures were not performed on the Day 3 samples
