# Supplementary material for: Gut microbiota markers in early childhood are linked to farm living, pets in household and allergy
Source: PLoS One. 2024 Nov 27;19(11):e0313078. doi: 10.1371/journal.pone.0313078 (PMC11602077; doi:10.1371/journal.pone.0313078)
Supplement: S4 Table — (DOCX) [file pone.0313078.s004.docx]

**S4 Table.** Bacterial variables associated with farm living, unadjusted and adjusted for pets in household, sex, and breastfeeding (proportion of days of any breastfeeding up to sampling).

|  |  | **Percent difference in colonization rate (95% CI)**  **Infants in farming vs non-farming families** | | |  |
| --- | --- | --- | --- | --- | --- |
| **Variable / age at sampling** | **n (%) colonized in farm/control** | **Unadjusted** | **Adjusted** | | **Higher (↑) / Lower (↓)**  **in farm group** |
| *C. difficile* colonization at 12 mo | 3 (11) / 18 (50) | -40 (-61 to -19)  p<0.001 | -41 (-65 to -18)  p<0.001 | | ↓ |
| CoNS colonization at 4 mo | 26 (93) /28 (78) | +15 (-2 to +32)  p=0.086 | +21 (+2 to +40)  p=0.029 | | ↑ |
| CoNS colonization at 12 mo | 21 (75) / 33 (92) | -17 (-36 to +2)  p=0.088 | -26 (-47 to -5)  p=0.014 | | ↓ |
| *S. aureus* colonization at 18 mo | 3 (12) / 12 (33) | -21 (-41 to 0)  p=0.046 | -18 (-39 to +3)  p=0.094 | | ↓ |
|  |  | **Fold change in population counts in colonized children (95% CI)**  **Infants in farming vs non-farming families** | | |  |
|  |  | **Unadjusted** | | **Adjusted** |  |
| Ratio anaerobe/facultative at 1 w | - | 11 (2.8 to 41)  p<0.001 | 7.0 (1.4 to 36)  p=0.020 | | ↑ |
| *E. coli* counts at 1 w | 17 (61) / 20 (61) | 0.07 (0.01 to 0.46)  p=0.006 | 0.09 (0.01 to 0.64)  p=0.017 | | ↓ |
| *E. coli* counts at 1 mo | 19 (68) /26 (70) | 0.22 (0.01 to 1.00)  p=0.049 | 0.15 (0.03 to 0.83)  p=0.030 | | ↓ |
| *E. coli* counts at 4 mo | 26 (93) / 31 (86) | 0.25 (0.06 to 1.1)  p=0.073 | 0.20 (0.05 to 0.78)  p=0.021 | | ↓ |
| *Bifidobacterium* counts at 6 mo | 25 (89) / 29 (78) | 3.6 (0.92 to 14)  p=0.066 | 6.0 (1.2 to 31)  p=0.032 | | ↑ |
| *Lactobacillus* counts at 6 mo | 21 (75) / 19 (51) | 32 (1.2 to >100)  p=0.040 | 70 (1.6 to >100)  p=0.027 | | ↑ |
| Statistical analyses were performed using generalized estimating equations (GEE) to account for intra-individual correlations in repeated measures data. Results are presented as differences in colonization rates and population counts in colonized children of bacterial variables associated with farm living, unadjusted and adjusted for potential confounders, with 95% confidence intervals (CIs). | | | | | |
