# Supplementary material for: Gut microbiota markers in early childhood are linked to farm living, pets in household and allergy
Source: PLoS One. 2024 Nov 27;19(11):e0313078. doi: 10.1371/journal.pone.0313078 (PMC11602077; doi:10.1371/journal.pone.0313078)
Supplement: S5 Table — (DOCX) [file pone.0313078.s005.docx]

**S5 Table.** Bacterial variables associated with pets in household, unadjusted and adjusted for farm living, sex, and breastfeeding (proportion of days of any breastfeeding up to sampling).

|  |  | **Percent difference in colonization rate (95% CI)**  **Infants in households with pets vs no pets** | |  |
| --- | --- | --- | --- | --- |
| **Variable / age at sampling** | **n (%) colonized in pets/no pets** | **Unadjusted** | **Adjusted** | **Higher (↑) / Lower (↓)**  **in pets group** |
| *Bifidobacterium* colonization at 2 w | 32 (91) / 17 (71) | +22 (+1 to +43) p=0.039 | +25 (+4 to +47) p=0.022 | ↑ |
| *Bacteroides* colonization at 1 mo | 22 (55) / 7 (29) | +26 (+1 to +51) p=0.041 | +24 (-2 to +51) p=0.072 | ↑ |
| *Bacteroides* colonization at 4 mo | 28 (72) / 8 (35) | +38 (+13 to +62) p=0.003 | +39 (+15 to +64) p=0.002 | ↑ |
| *Lactobacillus* colonization at 1 mo | 29 (73) / 11 (46) | +27 (+1 to +52) p=0.039 | +27 (+1 to +53) p=0.045 | ↑ |
| CoNS colonization at 2 mo | 30 (77) / 23 (96) | -19 (-35 to -3) p=0.021 | -18 (-38 to +2) p=0.071 | ↓ |
| CoNS colonization at 4 mo | 31 (78) / 22 (96) | -18 (-34 to 2) p=0.025 | -21 (-42 to -1) p=0.044 | ↓ |
| CoNS colonization at 12 mo | 36 (90) / 18 (78) | +12 (-8 to +32) p=0.25 | +21 (+1 to +41) p=0.036 | ↑ |
|  |  | **Fold change in population counts in colonized children (95% CI)**  **Infants in households with pets vs no pets** | |  |
|  |  | **Unadjusted** | **Adjusted** |  |
| Ratio anaerobe/facultative at 1 w | - | 7.2 (1.7 to 31) p=0.007 | 3.4 (0.83 to 14) p=0.088 | ↑ |
| *C. difficile* counts at 2 mo | 6 (15) / 2 (8) | 53 (1.8 to >100) p=0.023 | 39 (0.00 to >100) p=0.53 | ↑ |
| *E. coli* counts at 1 w | 24 (65) / 12 (52) | 0.19 (0.04 to 0.98) p=0.048 | 0.40 (0.07 to 2.2) p=0.30 | ↓ |
| *Enterococcus* counts at 2 mo | 38 (97) / 23 (96) | 10 (1.3 to 75) p=0.025 | 8.0 (1.2 to 56) p=0.036 | ↑ |
| CoNS counts at 12 mo | 36 (90) / 18 (78) | 3.6 (1.0 to 12) p=0.045 | 4.6 (1.2 to 17) p=0.024 | ↑ |
| Statistical analyses were performed using generalized estimating equations (GEE) to account for intra-individual correlations in repeated measures data. Results are presented as differences in bacterial colonization rates and population counts in colonized children of bacterial variables associated with pets in household, unadjusted and adjusted for potential confounders, with 95% confidence intervals (CIs). | | | | |
