# Supplementary material for: Gut microbiota markers in early childhood are linked to farm living, pets in household and allergy
Source: PLoS One. 2024 Nov 27;19(11):e0313078. doi: 10.1371/journal.pone.0313078 (PMC11602077; doi:10.1371/journal.pone.0313078)
Supplement: S6 Table — (DOCX) [file pone.0313078.s006.docx]

**S6 Table.** Bacterial variables associated with allergy at 3 years of age, unadjusted and adjusted for farm living, pet exposure, sex, breastfeeding (proportion of days of any breastfeeding up to sampling) and heredity (allergic parent(s)).

|  |  | **Percent difference in colonization rate (95% CI)**  **Allergy vs no allergy at 3 years of age** | |  |
| --- | --- | --- | --- | --- |
| **Variable / age at sampling** | **n (%) colonized in allergic/healthy** | **Unadjusted** | **Adjusted** | **Higher (↑) / Lower (↓)**  **in allergic children** |
| *Bacteroides* colonization at 1 w | 1 (10) / 25 (50) | -36 (-62 to -11) p=0.005 | -33 (-63 to -3) p=0.031 | ↓ |
| *Bacteroides* colonization at 2 w | 1 (10) / 23 (48) | -34 (-60 to -8) p=0.011 | -29 (-60 to +3) p=0.073 | ↓ |
| *Bacteroides* colonization at 1 mo | 2 (18) / 26 (50) | -32 (-61 to -3) p=0.030 | -23 (-57 to 11) p=0.19 | ↓ |
| *Bifidobacterium* colonization at 2 m | 5 (46) / 47 (92) | -47 (-80 to -14) p=0.006 | -39 (-75 to -3) p=0.032 | ↓ |
| *Bifidobacterium* colonization at 4 m | 5 (50) / 48 (92) | -43 (-77 to -8) p=0.017 | -35 (-72 to +2) p=0.067 | ↓ |
| *Lactobacillus* colonization at 4 mo | 2 (20) / 37 (71) | -48 (-79 to -17) p=0.003 | -54 (-88 to -19) p=0.002 | ↓ |
| *Lactobacillus* colonization at 6 mo | 3 (27) / 36 (69) | -42 (-74 to -10) p=0.010 | -34 (-72 to 3) p=0.072 | ↓ |
| *C. difficile* colonization at 4 mo | 6 (60) / 9 (17) | +43 (+8 to +78) p=0.016 | +39 (+1 to +78) p=0.043 | ↑ |
| *Clostridium* colonization at 4 mo | 10 (100) / 3 (6) | +23 (+11 to +36) p<0.001 | +20 (+2 to +38) p=0.033 | ↑ |
| CoNS colonization at 12 mo | 10 (100) / 43 (83) | +17 (+7 to +28) p=0.002 | +21 (+4 to +38) p=0.017 | ↑ |
| CoNS colonization at 18 mo | 10 (91) / 37 (74) | +17 (-6 to +39) p=0.14 | +29 (+2 to +56) p=0.036 | ↑ |
| *Enterococcus* colonization at 4 mo | 10 (100) / 47 (90) | +10 (+2 to +18) p=0.020 | +5 (-3 to +14) p=0.22 | ↑ |
| *E. coli* colonization at 18 mo | 11 (100) / 46 (92) | +8 (0 to +16) p=0.044 | +12 (-4 to +28) p=0.15 | ↑ |

|  |  | **Fold change in population counts in colonized children (95% CI)**  **Allergy vs no allergy at 3 years of age** | |  |
| --- | --- | --- | --- | --- |
|  | **n (%) colonized in allergic/healthy** | **Unadjusted** | **Adjusted** |  |
| Ratio anaerobe/facultative at 1 w | - | 0.06 (0.01 to 0.32) p=0.001 | 0.18 (0.02 to 1.3) p=0.094 | ↓ |
| *S. aureus* counts at 6 mo | 6 (55) / 25 (48) | 3.9 (0.36 to 41) p=0.26 | 15 (1.1 to >100) p=0.039 | ↑ |
| *Bacteroides* counts at 1 mo | 2 (18) / 26 (50) | 6.7 (2.3 to 20) p<0.001 | 6.4 (0.37 to >100) p=0.20 | ↑ |
| *Bacteroides* counts at 12 mo | 8 (80) / 49 (94) | 2.8 (0.87 to 8.6) p=0.083 | 3.4 (1.00 to 12) p=0.050 | ↑ |
| *Lactobacillus* counts at 2 mo | 8 (73) / 34 (67) | 6.3 (1.04 to 38) p=0.045 | 3.9 (0.26 to 57) p=0.32 | ↑ |
| *Lactobacillus* counts at 18 mo | 6 (55) / 34 (68) | 38 (2.7 to >100) p=0.008 | 15 (0.39 to >100) p=0.14 | ↑ |
| Non-*E. coli* counts at 2w | 2 (20) / 16 (33) | 29 (1.1 to >100) p=0.042 | 427 (0.00 to >100) p=0.30 | ↑ |
| Statistical analyses were performed using generalized estimating equations (GEE) to account for intra-individual correlations in repeated measures data. Results are presented as differences in bacterial colonization rates and population counts in colonized children of bacterial variables associated with allergy at 3 years of age, unadjusted and adjusted for potential confounders, with 95% confidence intervals (CIs). | | | | |
