# Supplementary material for: Gut microbiota markers in early childhood are linked to farm living, pets in household and allergy
Source: PLoS One. 2024 Nov 27;19(11):e0313078. doi: 10.1371/journal.pone.0313078 (PMC11602077; doi:10.1371/journal.pone.0313078)
Supplement: S7 Table — (DOCX) [file pone.0313078.s007.docx]

**S7 Table.** Bacterial variables associated with allergy at eight years of age, unadjusted and adjusted for farm living, pet exposure, sex, breastfeeding (proportion of days of any breastfeeding up to sampling) and heredity (allergic parent(s)).

|  |  | **Percent difference in colonization rate (95% CI)**  **Allergy vs no allergy at 8 years of age** | |  |
| --- | --- | --- | --- | --- |
| **Variable / age at sampling** | **n (%) colonized**  **in allergic/healthy** | **Unadjusted** | **Adjusted** | **Higher (↑) / Lower (↓)**  **in allergic children** |
| *Bacteroides* colonization at 1 w | 0 (0) / 19 (54) | -55 (-71 to -38) p<.0001 | -48 (-71 to -26) p<0.0001 | ↓ |
| *Bacteroides* colonization at 2 w | 0 (0) / 18 (51) | -52 (-69 to -36) p<.0001 | -45 (-69 to -21) p<0.001 | ↓ |
| *Bacteroides* colonization at 1 mo | 1 (10) / 19 (50) | -40 (-66 to -14) p=0.003 | -45 (-68 to -23) p<0.001 | ↓ |
| *Bifidobacterium* colonization at 1 w | 4 (40) / 27 (77) | -38 (-74 to -1) p=0.045 | -43 (-85 to -1) p=0.046 | ↓ |
| *Bifidobacterium* colonization at 2 w | 5 (50) / 31 (89) | -40 (-76 to -3) p=0.032 | -42 (-79 to -5) p=0.026 | ↓ |
| *Lactobacillus* colonization at 6 mo | 3 (30) / 25 (66) | -36 (-71 to 0) p=0.047 | -24 (-68 to 20) p=0.29 | ↓ |
| *Lactobacillus* colonization at 18 mo | 9 (90) / 21 (55) | +35 (+8 to +61) p=0.010 | +47 (+8 to +86) p=0.017 | ↑ |
| *C. difficile* colonization at 2 w | 0 (0) / 5 (14) | -14 (-26 to -2) p=0.019 | -15 (-30 to -0) p=0.049 | ↓ |
| *C. difficile* colonization at 6 mo | 7 (70) / 10 (26) | +44 (+9 to +78) p=0.014 | +38 (+6 to +69) p=0.019 | ↑ |
| *C. difficile* colonization at 12 mo | 5 (56) / 10 (26) | +31 (-9 to +71) p=0.13 | +39 (0 to +77) p=0.049 | ↑ |
| *Clostridium* colonization at 6 mo | 10 (100) / 32 (84) | +16 (+4 to +28) p=0.010 | +14 (-1 to +29) p=0.065 | ↑ |
| Non*-E. coli* colonization at 3 d | 0 (0) / 4 (11) | -11 (-22 to -1) p=0.035 | -11 (-25 to +2) p=0.091 | ↓ |
| Non*-E. coli* colonization at 18 mo | 1 (10) / 20 (53) | -43 (-69 to -16) p=0.002 | -46 (-88 to -5) p=0.028 | ↓ |
| *Enterococcus* colonization at 2 w | 9 (90) / 28 (80) | +12 (-13 to +37) p=0.34 | +21 (+5 to +38) p=0.012 | ↑ |
| *S. aureus* colonization at 6 mo | 8 (80) / 17 (45) | +35 (+3 to +67) p=0.031 | +17 (-19 to +54) p=0.35 | ↑ |
|  |  | **Fold change in population counts in colonized children (95% CI)**  **Allergy vs no allergy at 8 years of age** | |  |
|  | **n (%) colonized**  **in allergic/healthy** | **Unadjusted** | **Adjusted** |  |
| Ratio anaerobe/facultative at 1 w | - | 0.06 (0.01 to 0.36) p=0.002 | 0.06 (0.01 to 0.48) p=0.007 | ↓ |
| Ratio anaerobe/facultative at 2 w | - | 0.11 (0.02 to 0.74) p=0.024 | 0.12 (0.01 to 0.97) p=0.047 | ↓ |
| *S. aureus* counts at 1 mo | 7 (70) / 14 (37) | 151 (5.5 to >1000) p=0.003 | 133 (0.39 to >1000) p=0.099 | ↑ |
| *E. coli* counts at 12 mo | 9 (100) / 37 (97) | 5.1 (1.6 to 16) p=0.007 | 4.0 (0.66 to 24) p=0.13 | ↑ |
| *Clostridium* counts at 2 mo | 8 (80) / 22 (58) | 12 (1.6 to 86) p=0.014 | 14 (0.34 to >100) p=0.16 | ↑ |
| *Lactobacillus* counts at 4 mo | 5 (50) / 25 (68) | 0.03 (0.00 to 0.56) p=0.020 | 0.03 (0.00 to 1.8) p=0.091 | ↓ |
| *Bacteroides* counts 4 mo | 5 (50) / 18 (50) | 7.6 (1.2 to 51) p=0.036 | 4.8 (0.27 to 86) p=0.28 | ↑ |
| Statistical analyses were performed using generalized estimating equations (GEE) to account for intra-individual correlations in repeated measures data. Results are presented as differences in bacterial colonization rates and population counts in colonized children of bacterial variables associated with allergy at 8 years of age, unadjusted and adjusted for potential confounders, with 95% confidence intervals (CIs). | | | | |
