# Supplementary material for: Gut microbiota markers in early childhood are linked to farm living, pets in household and allergy
Source: PLoS One. 2024 Nov 27;19(11):e0313078. doi: 10.1371/journal.pone.0313078 (PMC11602077; doi:10.1371/journal.pone.0313078)
Supplement: S1 Appendix — (DOCX) [file pone.0313078.s008.docx]

**S1 Appendix. Culture media, growth conditions and identification methods used for the isolation and identification of bacterial groups that are commonly detected in the early gut microbiota.**

**Culture of rectal swabs**

Each rectal swab was streaked on four agar media: Colombia blood, Drigalski, Enterococcosel and Staphylococcus. Streaking was performed in a three step manner in order to yield free-lying colonies. The plates were incubated in air at 37^o^C for two days.

**Culture of fecal samples**

A volume of 170 ml of feces was diluted in 1.5 ml sterile peptone water (a 1/10 dilution, or -1 log dilution). Further tenfold dilutions were made to a -8 log dilution.

30 ml of each of the -1, -3, -5, -6, -7 and -8 dilutions were spread on the following types of agar media: Colombia blood, Drigalski, Enterococcosel, Staphylococcus, Brucella blood, Bacteroides Bile Esculin, Beerens, Rogosa and CCFA. For CCFA, only the log dilutions -1, -3, and -5 were cultured. The plates were incubated in air, or in an anaerobic atmosphere, as specified in the table below.

For clostridia (anaerobic spore-formers), vegetative bacterial cells were killed off, and the resulting suspension, containing spores, was cultured quantitatively to determine the clostridial spore count. To achieve this, 200 ml of the -1 dilution (1/10) was mixed with 200 ml of 99% ethanol and the mixture was incubated with shaking at room temperature for 30 min. This procedure kills living bacteria, but spores are resistant to ethanol. Thereafter, 200 ml of the mixture was diluted in 800 ml sterile peptone water and further diluted in 10-fold steps to -5. 30 ml of -1, -3 and -5 dilutions were spread on Brucella blood agar and incubated anaerobically, as specified in the table below.

*Culture media, growth conditions and identification methods used for the isolation and identification of the bacterial groups studied.*

| **Bacteria** | **Incubation condition** | **Time (days)** | **Medium** | **Reference** | **Method of identification** |
| --- | --- | --- | --- | --- | --- |
| Total facultatives | Air | 2 | Colombia blood agar | [1] | Aerobic growth |
| *Enterobacteriaceae* | Air | 2 | Drigalski agar | [2] | Gram-stain  API 20E biotyping system (Bio-Merieux, Marcy l'Etoile, France) |
| *Staphylococcus* | Air | 2 | Staphylococcus agar | [3] | Gram-stain  Coagulase test |
| *Enterococcus* | Air | 2 | Enterococcosel agar | [4] | Gram-stain  Esculin test |
| Total anaerobes^a^ | Anaerobic | 3 | Brucella blood agar | [5] | Anaerobic but not aerobic growth |
| *Bacteroides* | Anaerobic | 3 | Bacteroides bile esculin agar | [6] | Gram-stain  Rapid ID 32 A biotyping system (Bio-Merieux). |
| *Bifidobacterium* | Anaerobic | 3 | Beerens agar | [7] | Gram-stain  Genus-specific Real-Time PCR assay |
| *Lactobacillus* | Anaerobic | 3 | Rogosa agar | [8] | Gram-stain  Multiplex group-specific PCR assay |
| *Clostridium* | Anaerobic | 3 | Brucella blood agar | [5] | Gram-stain  Rapid ID 32 A biotyping system (Bio-Merieux). |
| *Clostridioides difficile* | Anaerobic | 3 | CCFA agar | [9] | Gram-stain  Rapid ID 32 A biotyping  system (Bio-Merieux). |

Rectal swabs (aerobic culture only) and serially diluted fecal samples (quantitative aerobic and anaerobic cultures) were incubated at 37^o^C on the above-listed media for the enumeration of the indicated bacterial groups. The methods used for culturing and identification have previously been described [10-13].

**Determination of total bacterial counts**

The total concentration of facultative bacteria per gram of feces was calculated based on counting the colonies on an appropriate dilution cultured aerobically on Colombia blood agar. A dilution yielding 10 - 100 colonies was selected. The number of CFU/g feces was determined by first calculating the number of CFU/ml of the dilution, then dividing this number by the dilution. For example: if 85 colonies were identified on the -6 log dilution plate, the number of CFU/ml of the -6 log dilution would be 85 x 1/0.030 (since 30 ml of the dilution was spread on the plate), and the number of CFU/g feces would be 85 x 1/0.030 x 10^6^. This way of calculating CFU/g feces was applied for all determinations of bacterial counts.

The total concentration of anaerobic bacteria was calculated by determining the numbers growing on anaerobically incubated Brucella blood agar, from which the number of facultative bacteria was subtracted, as facultatives grow both in the presence and absence of air. Therefore, each colony type identified on anaerobically incubated Brucella blood agar was subcultured in air and isolates growing aerobically were not included in the total anaerobic counts. An exception to this rule was that weak growth of Gram-positive rods under aerobic conditions was accepted (as several species of *Lactobacillus* or *Bifidobacterium* may show scant growth under aerobic conditions).

**Isolation, Gram-staining and enumeration of distinct colony types**

All morphologically distinguishable colony types in a sample were isolated and enumerated as follows. For each type of agar plate, dilutions giving free-lying colonies of different colony types were selected. Each colony type was enumerated separately and its population count was determined. A representative colony of each colony type was picked, subcultured for purity on Colombia blood agar (for facultatives) or Brucella blood agar (for obligate anaerobes) and subjected to gram-staining <https://asm.org/getattachment/5c95a063-326b-4b2f-98ce-001de9a5ece3/gram-stain-protocol-2886.pdf>. The slides with Gram-stained bacterial smears were examined under the microscope and the color and shape of the bacterial cells (Color: Gram-positive, Gram-negative or Gram-variable, Shape: cocci or rods, small or large, bifid cells or cells with club shaped ends, presence of endospores) were noted for each isolate (“Gram-stained appearance”). The isolates were identified to the genus or species level (as described below), either directly or after storage at -70^o^C.

Certain bacteria (e.g. *Bifidobacterium*) may grow on several types of media. The population count of each bacterium was calculated based on its colony number on the selective agar plates intended for isolation of this type of bacteria, e.g. Beerens agar for *Bifidobacterium*. If the bacterium in question was not isolated from “its own” selective medium, but grew on media intended for the isolation and enumeration of other types of bacteria, or only on the non-selective medium, its presence was recorded, but its population counts were not calculated. Thus, the child was recorded as being colonized by this bacterium, but having missing data regarding the population counts of this bacterium.

**Bacterial identification**

*Enterobacteriaceae family*

Gram-negative rod shaped bacteria growing aerobically on Drigalski agar [2] were identified to the species or genus level using API 20E biotyping system (Bio-Merieux, Marcy l'Etoile, France) according to the manufacturer´s instructions. Bacteria belonging to the *Enterobacteriaceae* family were in most cases identified to the species level.

*Staphylococcus*

Gram-positive cocci growing aerobically on Staphylococcus agar [3] and giving a positive catalase reaction <https://asm.org/getattachment/72a871fc-ba92-4128-a194-6f1bab5c3ab7/Catalase-Test-Protocol.pdf> were identified as staphylococci. *Staphylococcus aureus* was distinguished from coagulase-negative staphylococci (CoNS) based on a tube coagulase test <https://asm.org/ASM/media/Protocol-Images/Coagulase-Test-Protocol.pdf?ext=.pdf> .

*Enterococcus*

Gram-positive cocci growing aerobically on Enterococcosel agar were identified as enterococci based on typical Gram-stained appearance and the ability to hydrolyze esculin on the Enterococcosel agar plates [4].

*Bacteroides*

Anaerobic Gram-negative rod-shaped bacteria isolated from Bacteroides Bile Esculin agar [6], or occasionally from non-selective Brucella blood agar [5], were identified as *Bacteroides* spp using Rapid ID 32 A biotyping system (Bio-Merieux, Marcy l'Etoile, France) according to the manufacturer´s instructions.

*Bifidobacterium*

Anaerobic Gram-positive rod-shaped bacteria isolated from Beerens agar [7], or occasionally from other types of media, were identified as *Bifidobacterium* spp. in a bifidobacterial genus-specific Real-Time PCR assay using previously published probe and primers [11]. Some suspected bifidobacterial isolates failed to grow on subculture (and could not be subjected to the PCR analysis), but were defined as *Bifidobacterium* if they exhibited typical bifid or club-shaped forms in the microscopic evaluation of the Gram-stained smear.

*Lactobacillus*

Anaerobic Gram-positive rod-shaped bacteria isolated from Rogosa agar plates [8] or occasionally from other types of media, were identified as *Lactobacillus* spp. in a *Lactobacillus* group-specific multiplex PCR assay using previously published primers [13] identifying 4 groups of *Lactobacillus* spp. (I-IV), where group I includes *L. delbrueckii*, group II *L. jensenii, L, acidophilus, L crispatus, L. gasseri*, *L. johnsonii*, *L. helveticus*and *L. amylovorus*, group III *L. paracasei* and *L. rhamnosus* and group IV *L. salivarius*, *L. reuteri*, *L. plantarum* and *L. fermentum* [12].

*Clostridium*

Anaerobic Gram-positive or Gram-variable rod-shaped bacteria isolated from Brucella blood agar plates seeded with dilutions of ethanol treated fecal samples (or occasionally from other agar plates seeded with sample dilutions not treated with alcohol) were identified as *Clostridium* spp using Rapid ID 32 A biotyping system (Bio-Merieux, Marcy l'Etoile, France) according to the manufacturer´s instructions. Some isolates of spore-forming Gram-positive or Gram-variable rod-shaped anaerobic bacteria failed to grow when subcultured, but were accepted as *Clostridium* spp. based on strict anaerobic growth and gram-stained appearance.

*Clostridioides difficile*

Gram-positive or Gram-variable rod-shaped bacteria yielding colonies with typical macroscopic appearance on CCFA agar plates cultured anaerobically [9], and occasionally isolates from other agar plates, were identified as *C. difficile* using Rapid ID 32 A biotyping system (Bio-Merieux, Marcy l'Etoile, France) according to the manufacturer´s instructions.

**References:**

1. Ellner PD, Stoessel CJ, Drakeford E, Vasi F. A new culture medium for medical bacteriology. Am J Clin Pathol. 1966;45(4):502-4.

2. Kauffman F. The bacteriology of Enterobacteriaceae 2nd Ed. Copenhagen: Munkgaard; 1969.

3. Chapman GH. Comparison of Ludlam's medium with Staphylococcus medium number 110 for the isolation of staphylococci that clot blood. J Bacteriol. 1949;58(6):823.

4. Isenberg HD, Goldberg D, Sampson J. Laboratory studies with a selective Enterococcus medium. Appl Microbiol. 1970;20(3):433-6.

5. Slots J. Bacterial specificity in adult periodontitis. A summary of recent work. J Clin Periodontol. 1986;13(10):912-7.

6. Livingston SJ, Kominos SD, Yee RB. New medium for selection and presumptive identification of the Bacteroides fragilis group. J Clin Microbiol. 1978;7(5):448-53.

7. Silvi S, Rumney CJ, Rowland IR. An assessment of three selective media for bifidobacteria in faeces. J Appl Bacteriol. 1996;81(5):561-4.

8. Rogosa M, Mitchell JA, Wiseman RF. A selective medium for the isolation and enumeration of oral and fecal lactobacilli. J Bacteriol. 1951;62(1):132-3.

9. George WL, Sutter VL, Citron D, Finegold SM. Selective and differential medium for isolation of Clostridium difficile. J Clin Microbiol. 1979;9(2):214-9.

10. Adlerberth I, Lindberg E, Aberg N, Hesselmar B, Saalman R, Strannegard IL, et al. Reduced enterobacterial and increased staphylococcal colonization of the infantile bowel: an effect of hygienic lifestyle? Pediatr Res. 2006;59(1):96-101.

11. Penders J, Vink C, Driessen C, London N, Thijs C, Stobberingh EE. Quantification of Bifidobacterium spp., Escherichia coli and Clostridium difficile in faecal samples of breast-fed and formula-fed infants by real-time PCR. FEMS Microbiol Lett. 2005;243(1):141-7.

12. Ahrne S, Lonnermark E, Wold AE, Aberg N, Hesselmar B, Saalman R, et al. Lactobacilli in the intestinal microbiota of Swedish infants. Microbes Infect. 2005;7(11-12):1256-62.

13. Song Y, Kato N, Liu C, Matsumiya Y, Kato H, Watanabe K. Rapid identification of 11 human intestinal Lactobacillus species by multiplex PCR assays using group- and species-specific primers derived from the 16S-23S rRNA intergenic spacer region and its flanking 23S rRNA. FEMS Microbiol Lett. 2000;187(2):167-73.
